# Efficacy of a transdiagnostic ecological momentary intervention for improving self-esteem (SELFIE) in youth exposed to childhood adversity: study protocol for a multi-center randomized controlled trial

**DOI:** 10.1186/s13063-021-05585-y

**Published:** 2021-09-19

**Authors:** Maud Daemen, Mary Rose Postma, Ramon Lindauer, Iris Hoes-van der Meulen, Dorien Nieman, Philippe Delespaul, Josefien Johanna Froukje Breedvelt, Mark van der Gaag, Wolfgang Viechtbauer, Koen Schruers, David van den Berg, Claudi Bockting, Therese van Amelsvoort, Ulrich Reininghaus

**Affiliations:** 1 Department of Psychiatry and Neuropsychology, School for Mental Health and Neuroscience, Maastricht University, Maastricht, The Netherlands; 2 Department of Public Mental Health, Central Institute of Mental Health, Medical Faculty Mannheim, University of Heidelberg, Mannheim, Germany; 3 Mondriaan, Maastricht, The Netherlands; 4 Department of Child and Adolescent Psychiatry, Amsterdam UMC, location Academic Medical Center, Amsterdam, The Netherlands; 5 Levvel, academic centre for child and adolescent psychiatry, Amsterdam, The Netherlands; 6 Prodeba Mental Health Care, Leiden, The Netherlands; 7 Department of Psychiatry, Amsterdam University Medical Centers (location AMC), Amsterdam, The Netherlands; 8 Centre for Urban Mental Health, University of Amsterdam, Amsterdam, The Netherlands; 9 Department of Clinical Psychology, VU University, Amsterdam, The Netherlands; 10 Parnassia Academy, The Hague, The Netherlands; 11 Department of Health Psychology, Faculty of Psychology, University of Leuven, Leuven, Belgium; 12 Centre for Epidemiology and Public Health, Health Service and Population Research Department, Institute of Psychiatry, Psychology & Neuroscience, King’s College London, London, UK

**Keywords:** SELFIE, Ecological momentary intervention, mHealth, Experience Sampling Method, Self-esteem, Childhood trauma, Childhood adversity

## Abstract

**Background:**

Targeting low self-esteem in youth exposed to childhood adversity is a promising strategy for preventing adult mental disorder, but psychological help remains difficult to access and accept for youth, calling for novel, youth-friendly approaches. Mobile Health (mHealth) and, most prominently, ecological momentary interventions (EMIs) provide a unique opportunity to deliver youth-friendly, personalized, real-time, guided self-help interventions. The aim of this study is to investigate the efficacy of a novel, accessible, transdiagnostic ecological momentary intervention for improving self-esteem (‘SELFIE’) in youth with prior exposure to childhood adversity.

**Methods/design:**

In a parallel-group, assessor-blind, multi-center randomized controlled trial, individuals aged 12–26 years with prior exposure to childhood adversity and low self-esteem will be randomly allocated to SELFIE in addition to treatment as usual (TAU) as the experimental condition or the control condition of TAU only, which will include access to all standard health care. SELFIE is a digital guided self-help intervention administered through a smartphone-based app to allow for interactive, personalized, real-time and real-world transfer of intervention components in individuals’ daily lives, blended with three training sessions delivered by trained mental health professionals over a 6-week period. Outcomes will be assessed at baseline, post-intervention, and 6-month follow-up by blinded assessors. The primary outcome will be the level of self-esteem as measured with the Rosenberg Self-Esteem Scale (RSES).

**Discussion:**

The current study is the first to establish the efficacy of an EMI focusing on improving self-esteem transdiagnostically in youth exposed to childhood adversity. If this trial provides evidence on the efficacy of SELFIE, it has significant potential to contribute to minimizing the deleterious impact of childhood adversity and, thereby, preventing the development of mental disorder later in life.

**Trial registration:**

Netherlands Trial Register NL7129 (NTR7475). Registered on 9 November 2018

## Introduction

The majority of mental disorders first emerge in youth and, as such, contribute substantially to disease burden, which is higher in youth than during any other developmental period [1–5]. More specifically, 50% of lifetime cases of mental disorder have started by age 14 years and three quarters by age 24 [2, 6]. Mental disorders in youth aged 10–24 years are associated with an immense cost [7–9] and have been found to be the leading cause of disease burden in high-income countries [4, 5]. Onset of a mental disorder may disrupt critical age-specific developmental, interpersonal, occupational, and educational milestones [10–12] and indicates a need for close scrutiny of the complex interplay between risk and protective factors in childhood and adolescence. Recently, transdiagnostic frameworks have become more prominent (e.g., the Hierarchical Taxonomy of Psychopathology (HiTOP) [13, 14], which broadly posit that symptoms of psychopathology are transdiagnostic in the early stages [15] and might result in a wide range of psychopathology later in life [12, 16]. Furthermore, during the ongoing COVID-19 pandemic, measures to control SARS-CoV-2 transmission rates have been shown to have negative effects on mental health, especially in youth [17–19]. All this highlights the value of transdiagnostic preventive interventions to improve well-being and resilience in youth and prevent morbidity later in life in order to reduce burden for individuals, families, and the wider society [2, 11, 20–22].

Youth referred to mental health services have experienced disproportionate levels of childhood adversity (i.e., abuse, neglect, bullying and household discord) [23–31], which is one of the most pervasive risk factors for developing a range of mental disorders [25, 32, 33]. For example, in a nation-wide Dutch study of help-seeking adolescents and young adults with an Ultra High Risk state for Psychosis (UHR), a high prevalence was found for physical (20.9%), sexual (24.8%), and emotional (46.7%) abuse, as well as physical (41.9%) and emotional (66.7%) neglect [29]. Also, in a study based on a representative sample drawn randomly from the general population in the Netherlands, it was shown that 29.7% experienced one or more adversities during their childhood [34]. Current estimates of attributable risks further suggest that interventions targeted at averting childhood adversity from exerting its adverse effects can prevent a substantial proportion of the incidence of adult mental disorder, and, thereby, have a sizeable public health impact and reduce societal costs [26, 35]. While primary prevention of childhood adversity through universal, population-based strategies is of prime importance, it remains difficult to achieve for all, and, hence, interventions targeting the negative psychological consequences of childhood adversity in youth are a promising selective prevention strategy for adverse outcomes later in life with tangible public health implications [31, 36].

One important psychological mechanism in pathways from childhood adversity to adult psychopathology is low self-esteem [37, 38]. Youth is a critical period for the development of self-esteem. Self-esteem is essential to well-being and mental health per se, with a substantial impact on the development and maintenance of severe mental disorders [39]. There is now substantial evidence to suggest that exposure to childhood adversity has detrimental effects on self-esteem [40–43]. The current evidence further suggests that childhood adversity exerts its detrimental effects on risk of later psychopathology precisely via pathways through low self-esteem [36, 37, 44–47]. The prevalence of low self-esteem in help-seeking youth has been reported to be around 45% [48]. Taken together, targeting low self-esteem at an early stage in youth exposed to childhood adversity is a promising strategy for preventing mental disorder and reducing societal costs.

Current psychological help, including prevention, however, remains difficult to access and accept for youth and has limited efficacy under real-world conditions, calling for novel approaches [49, 50]. While conventional interventions have proven efficacious in reducing psychiatric symptoms via enhancing self-esteem [51], a key next step is to develop and evaluate interventions that are specifically geared toward the specific needs of youth. This is what the current study is designed to achieve. The recent advances in information and communication technologies have led to the development of mobile Health (mHealth) interventions and, most prominently, ecological momentary interventions (EMIs) [52–56]. EMIs provide a unique opportunity to deliver youth-friendly, accessible, personalized, real-time, guided self-help interventions targeting candidate psychological mechanisms in daily life and thereby prevent mental disorder and reduce disease burden. This enables youth to access interventions that are individually adapted to their needs in a given moment and context (e.g., by offering interventions specifically tailored for helping participants in moments of low self-esteem). Recently, the term “Just-In-Time Adaptive Interventions (JITAIs)” has been started to be used by some authors [57, 58], positing that novel characteristics of JITAIs are that interventions are initiated by push notifications and dynamically initiated by the app. However, these features have been part of EMIs from the outset, and, hence, if anything JITAIs may be used synonymously with EMIs, which have been proposed at a much earlier point. EMIs are ideally placed for enhancing access to mental health services for youth depending on their needs and preferences by delivering low-threshold interventions by mental health professionals as one component that can be rolled out across child, adolescent and adult mental health services.

Previous studies of conventional interventions suggest that psychiatric symptoms, such as anxiety and depression symptoms, may be reduced through enhancing self-esteem [51]. However, these interventions are not tailored toward the specific preferences and needs of youth as naturally occurring in daily life. While EMIs such as the SELFIE intervention provide a unique opportunity to deliver youth-friendly, accessible, personalized, real-time interventions in daily life, robust trial-based evidence on EMIs and other mHealth interventions remains very limited [22, 52, 53, 59–62].

The overall aim of the current study is to test the efficacy of a novel, accessible, transdiagnostic ecological momentary intervention (EMI) for improving self-esteem (“SELFIE”) in youth aged 12–26 with prior exposure to childhood adversity in a multi-center randomized controlled trial (RCT). The SELFIE intervention will be administered in addition to treatment as usual (TAU) (experimental condition) and compared to a control condition of TAU only, which will include (access to) standard health care.

The specific objectives of this study are to:

1. Test the efficacy of the SELFIE intervention on improving self-esteem at post-intervention and 6-month follow-up (primary outcome);
2. Test the efficacy of the SELFIE intervention on improving momentary self-esteem, positive and negative schematic beliefs of self, resilience, emotional well-being, general psychopathology, functioning, and quality of life at post-intervention and 6-month follow-up (secondary outcomes);
3. Establish whether the effects of the SELFIE intervention on primary and secondary outcomes hold at 18-month and 24-month follow-up;
4. Examine the cost effectiveness and cost utility of the SELFIE intervention;
5. Assess the acceptability, safety, adherence and fidelity of the SELFIE intervention.

## Methods

### Study design

**Fig. 1:**
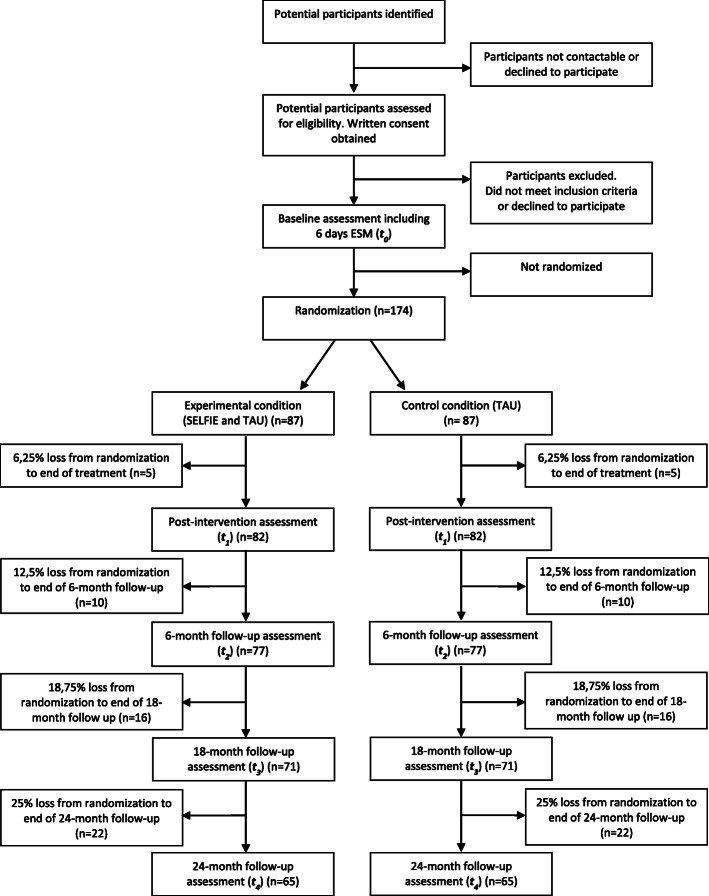
Overview of the study design

**Fig. 2:**
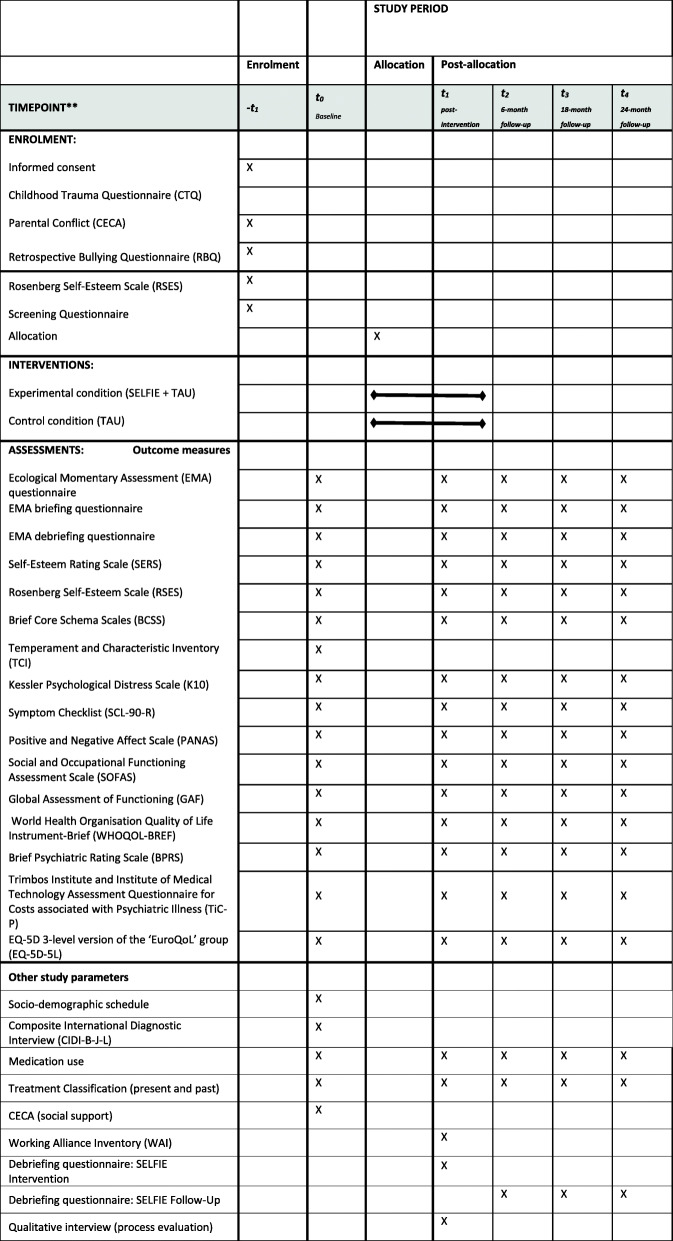
Standard Protocol Items: Recommendations for Interventional Trial (SPIRIT) figure. Ecological momentary intervention for improving self-esteem (SELFIE): schedule of enrolment, interventions, and assessments

In a two-arm parallel-group, assessor-blind multi-center randomized controlled trial, individuals aged between 12 and 26 years with prior exposure to childhood adversity and low self-esteem will be randomly allocated to SELFIE in addition to TAU as the experimental condition or a control condition of TAU only, which includes (access to) standard health care and social services. Participants will be recruited from mental health services in Noord-Holland, Zuid-Holland, and Limburg (the Netherlands) and from the general population (e.g., via social media). Outcomes will be measured at baseline (i.e., before randomization), post-intervention (i.e., after the 6-week intervention period), and 6-month, 18-month, and 24-month follow-up (i.e., 6, 18 and 24 months after completing the intervention period) by blind assessors (see Figs. 1 and 2). Randomization will be conducted independently of the research team through a computer-generated sequence, stratified by region of collaborating centers or as external admission. All outcomes will be measured and the statistical analysis will be performed blind to treatment allocation.

### Participants

A sample of 174 individuals aged 12–26 with prior exposure to childhood adversity and low self-esteem will be recruited. Participants will be recruited from collaborating mental health services in three regions in the Netherlands: Noord-Holland (Amsterdam University Medical Centers (Location AMC); Levvel), Zuid-Holland (Parnassia Group; Prodeba), and Limburg (Mondriaan; Lionarons GGZ; Koraalgroep). In addition, participants from the general population, who do not seek help from collaborating mental health services, will be recruited, e.g., via (targeted adverts on) social media, schools, social services, and flyers at relevant public locations. All individuals presenting to collaborating mental health services will be approached by their treating clinician, who will provide initial information about the study. If the individual is interested in the study, their treating clinician will, in agreement with the potential participant, pass on their contact details to the research team. All potential participants (including those recruited via social media, etc.) will be contacted by the research team and will be fully informed about the study. One week later, informed consent will be obtained (if applicable, also from parents/legal guardians), which can be withdrawn by participants at any time and without having any negative consequences for their access to standard health care). For participants under the age of 16, both parents (or the legal guardian) and participants will receive detailed information about the intervention. In addition, parents (or the legal guardian) and the researcher will consider possible negative reactions of the underage participant to the intervention procedure. Further, it will be determined together with the parents (or the legal guardian) prior to the intervention what the researcher will do in case of reluctance of the underage participant and which behavior of the participant commonly reflects reluctance. It will be discussed with the parents (or the legal guardian) when the study should be stopped in case of reluctance of their child.

Potential participants will then be asked to complete the screening questionnaires to assess whether they meet the inclusion criteria. Participants aged 16 years or older will be financially compensated for their time, and travel expenses will be fully reimbursed. To minimize loss to follow-up, researchers maintain contact with participants on a regular basis. Also, participants will receive a small additional financial reimbursement for completing all follow-up assessments.

#### Inclusion criteria

Inclusion criteria are as follows (see Table 1 for more detail): (1) aged between 12 and 26 years, (2) exposure to childhood adversity (physical, sexual, or emotional abuse, emotional or physical neglect, peer bullying, or parental conflict), (3) self-esteem below average measured with the Rosenberg Self-Esteem Scale (RSES) [51, 68, 69], (4) willingness to participate, (5) ability to give informed consent, and (6) parental consent for minors.

**Table 1:** Eligibility criteria

Inclusion criteria	
1) Aged between 12 and 26 years old.	
2) Adversity: a. Childhood trauma: Prior exposure to at least one form of childhood trauma defined as moderate or severe physical (score ≥ 10), sexual (score ≥ 8) and/or emotional (score ≥ 13) abuse, emotional (score ≥ 15), and/or physical (score ≥ 10) neglect, according to established severity categories of the Childhood Trauma Questionnaire (CTQ) [63–65], and/or b. Peer bullying: Exposure to moderate or severe peer bullying, measured with the Retrospective Bully Questionnaire (RBQ) (score of frequency of bullying in one or more ways “sometimes” or more often and/or classified the experience as “quite serious” or “extremely serious”) [66], and/or c. Parental conflict: A score of moderate or severe parental conflict, measured with the Childhood Experiences of Care and Abuse Questionnaire (CECA.Q) section Parental Conflict (frequency score of “regularly” or “often” and/or a severity score of “serious” or “violence”) [67].	
3) Self-esteem below average (measured with the Rosenberg Self-Esteem Scale (RSES) (score < 26) [51, 68].	
4) Willingness to participate in the SELFIE intervention.	
5) Ability to give written informed consent.	
6) Parental consent for minors.	
Exclusion criteria	
1) Insufficient command of Dutch	
2) Psychiatric symptoms due to an organic cause	

#### Exclusion criteria

Subjects will be excluded if their command of Dutch is insufficient or if their psychiatric symptoms are due to an organic cause.

### Intervention

#### Control condition: treatment as usual (TAU)

Participants allocated to the control condition will receive treatment as usual (TAU), which will include access to all standard health care and social services. Specifically, this will include all the input from their general practitioner and other providers of health and social services that they would receive if they did not participate in the study, except for manualized treatment that explicitly addresses self-esteem as primary target (e.g., COMET or EMDR [51, 70, 71]) during the intervention period.

#### Experimental condition: SELFIE + TAU

Participants allocated to the experimental condition will receive the manualized SELFIE intervention within a 6-week period in addition to TAU. The intervention consists of three face-to-face sessions, each for around 60 min, delivered by SELFIE therapists, who will be trained mental health professionals (e.g., psychologists, social workers and mental health nurse specialists trained in the SELFIE intervention and receiving regular supervision and inter-vision led by a clinical psychologist), three e-mail contacts, and an EMI administered through a smartphone-based app (i.e., the PsyMate® app) for adaptive real-time and real-world transfer of intervention components tailored to person, moment, and context, delivered over a 6-week intervention period. Due to the COVID-19 pandemic, some of the face-to-face sessions will be offered through a secure and encrypted video conferencing system. The intervention is based on principles of EMIs [22, 52–56, 62, 71], and a guided self-help approach using principles of cognitive-behavioral therapy (CBT), aimed at modifying cognitive bias inherent to negative self-esteem and developing and practicing a new behavioral repertoire guided by therapists using modeling and shaping as additional important therapeutic techniques [72, 73]. Delivering the intervention in individuals’ daily lives, and enabling youth to benefit from this intervention in a given moment and context, when most needed (e.g., in moments of low self-esteem) is the key goal of the 6-week SELFIE intervention. Therefore, in the first introductory session, participants will either receive a study smartphone with the app already installed or will be asked to install it on their own smartphone by the SELFIE therapist, who will explain the SELFIE intervention in detail and ask the participant to complete examples of training tasks on the app in order to address the self-selected goals the participant wants to work on in the 6-week intervention period. The app will offer participants “enhancing,” “consolidating,” and “interactive” tasks (see Table 2) [72, 73]. In enhancing tasks, new intervention components will be introduced and practiced, some of which will be modified and extended over the study period. Consolidating tasks will ask participants to practice previously learned components of enhancing tasks on a daily basis. For these tasks, participants will be reminded by the app between 1 and 3 times per day (varying by intervention week). During the intervention period, the Experience Sampling Method (ESM), a structured dairy technique, will be used to assess momentary self-esteem, affect, and pleasantness of activities and events, six times a day, on days 3, 4, and 5 in each of the six intervention weeks using a time-based design with stratified random sampling (i.e., with ESM assessments scheduled at random within set blocks of time) to allow for interactive tasks. Interactive tasks will be provided based on their ESM ratings of (positive and negative) affect, momentary self-esteem and pleasantness of activities and events. For example (in week 1), participants will be provided with an interactive task, offering them to add more successes to their positive datalog when they scored high on positive affect, momentary self-esteem and/or pleasantness of activities. Participants can discontinue the intervention at any time upon request without negative consequences.

**Table 2:** Key components of the SELFIE intervention

SELFIE intervention (weeks)						
	1	2	3	4	5	6
**Training session**	Face-to-face session 1	E-mail contact 1	Face-to-face session 2	E-mail contact 2	Face-to-face session 3	E-mail contact 3
**Enhancing EMI tasks**	Formulating a new positive core belief + Positive datalog (enter daily successes)	Personal positive qualities (integrated in positive datalog) + Tips to identify more positive qualities + One-minute exercise (listing (previously identified) positive qualities)	Overview old behavioral patterns + Development of new behavior patterns	Expanding the positive datalog with successes arising from new behavioral patterns	Strategies to deal with criticism + A critical look at criticism + Cost-benefit analysis of perfectionism + The minimum programme (practicing to perform less than perfect)	Writing a positive story about yourself + Maintenance plan (for after the intervention)
**Consolidating EMI tasks**	Positive datalog + Tips to add more successes in the positive datalog + Rating credibility of the new core belief	Positive datalog + Tips positive datalog + One-minute exercise + Rating credibility of the new core belief	Positive datalog + One-minute exercise + Rating credibility of the new core belief	Positive datalog + One-minute exercise + Expanding new behavior patterns + Rating credibility of the new core belief	Positive datalog + One-minute exercise + A critical look at criticism + Rating credibility of the new core belief	Positive datalog + One-minute exercise + Rating credibility of the new core belief
**Interactive EMI tasks**	Positive datalog (adding successes) Or Positive datalog (viewing previously identified successes)		Positive datalog (adding successes and/or positive qualities) Or Positive datalog (viewing previously identified successes and qualities)		A critical look at criticism	

See Postma [73] and De Neef [72] for more details

#### Outcome measures

After obtaining written informed consent and eligibility assessment, participants will complete a range of self-report, interview-based and computer-based measures to assess primary and secondary outcomes and other study parameters. Participants will complete self-report questionnaires using a smartphone-based app (i.e., the PsyMate® app). Interviews will be conducted using a secure and encrypted video conferencing system. In addition, ESM data will be collected following the protocol from previous ESM studies using the PsyMate® app to measure momentary self-esteem, emotional well-being, stress sensitivity, threat anticipation, and psychotic experiences in daily life for a period of 6 consecutive days [22, 31, 62, 74–76]. On each day, participants will be asked eight times per day to complete an ESM, which will be scheduled at random within set blocks of time. At the end of the 6-day baseline ESM period, participants will be asked to complete a short debriefing questionnaire. All the above-mentioned measures will be assessed at baseline (i.e., before randomization), post-intervention (i.e., after the 6-week intervention period), and 6-month follow-up. Please see Fig. 2 (SPIRIT Figure) for details of assessment at each time point. All assessments will be checked for quality and completeness by another member of the research team and an extensive data checking and cleaning will be adhered to as a quality control measure.

#### Primary outcome

The primary outcome will be global self-esteem, measured with the Rosenberg Self-Esteem Scale (RSES) [69], which is a widely used measure to assess global self-esteem with good reliability and validity [68, 77]. The RSES consists of ten items rated on a 4-point Likert scale ranging from “strongly agree” to ”strongly disagree”. The level of global self-esteem, operationalized as the total score of the RSES, will be compared between the experimental and the control condition at post-intervention and 6-month follow-up (H1).

#### Secondary outcomes

Secondary outcomes will include the level of momentary, positive and negative self-esteem, resilience, emotional well-being, positive and negative schematic beliefs of self, psychological distress, functioning, subjective quality of life, general psychopathology, clinical symptoms and health-related quality of life, service use (including admission to inpatient services) and cost, which will be compared between the experimental and control condition at post-intervention and at 6-month follow-up (H2). In addition, all secondary outcomes (incl. levels of global self-esteem, operationalized using the total score of the RSES (see previous section)) will be compared between the experimental and control condition and at 18- and 24-month follow-up (H3).

Momentary self-esteem will be assessed with four items, rated on a 7-point scale, using the ESM [78, 79]. The mean score will be used for analysis. Positive and negative self-esteem will be measured with the SERS, which is a 20-item rating scale to assess these two dimensions of self-esteem separately with good reliability and validity [80]. The total sum score of the positive dimension and the total sum score of the negative dimension will be used in the analysis. Momentary resilience will be assessed with the ESM positive affective recovery from event-related stress in daily life (operationalized as the return to baseline levels of positive affect following event-related stress) [31, 74, 76, 81]. We will assess emotional well-being using the Positive and Negative Affect Scale (PANAS) [82] based on the total sum score of the negative affect items and the total sum score of the positive affect items. Also, a 5-item ESM measure will be used for assessing negative affect and a 4-item ESM measure of positive affect [31, 74, 83]. For both measures, a mean score will be used in the analysis. The Brief Core Schema Scale (BCSS) will be used as an established measure of positive and negative schematic beliefs of self and others [84]. The following four total scores (all consisting of six items) will be obtained for use in the analysis: negative-self, positive-self, negative-others, and positive-others. Psychological distress will be measured with the Kessler Psychological Distress Scale (K10), which is widely used and well-validated in youth [85, 86]. A total sum score ranging from 10 to 50 will be used for analysis.

The Social and Occupational Functioning Assessment Scale (SOFAS) [87] and the Global Assessment of Functioning (GAF) scale [88] will be used as a well-validated measure of functioning in youth [86]. The overall level of functioning rated by researchers on a scale of 0 to 100 will be used in the analysis.

Subjective quality of life will be measured with the World Health Organization Quality of Life Instrument-Brief (WHOQOL-BREF) [86, 89]. Mean scores of all four domains (physical health, psychological, social relationships, environment) will be used. The revised Symptom Checklist (SCL-90-R) will be used as a reliable and valid measure to assess general psychopathology in youth [86, 90]. The measure consists of 90 items, which will be rated on a 5-point scale. The total sum score of the SCL-90-R will be used for analysis. We will use the 24-item version of the Brief Psychiatric Rating Scale (BPRS) [91, 92] as a validated interviewer measure to assess clinical symptoms of psychopathology in youth [86]. All items are rated on a 7-point scale and, for the analysis, the BPRS total score will be computed.

The Trimbos Institute and Institute of Medical Technology Assessment Questionnaire for Costs associated with Psychiatric Illness (TiC-P) [93] will be used to collect data on service use (including admission to inpatient services) and cost for cost-effectiveness analysis. Last, data on health-related quality of life will be operationalized by quality-adjusted life years (QALYs), which will be calculated based on the EQ-5D 5-level version of the “EuroQoL” group (EQ-5D-5L) for cost-utility analysis [94].

#### Process evaluation

A process evaluation will be performed following the methodology of realist evaluation [95]. Initial program theories will be developed based on transcribed data from a focus group with stakeholders as well as expert interviews. Overarching program theory and accompanying context-mechanism-outcome configurations will be tested among intervention users (individual interviews with participants who have completed the SELFIE intervention) as well as those who deliver the intervention (focus group with SELFIE therapists), through iterative data collection. Atlas.Ti will be used as software to support the process of our analyses.

#### Acceptability, adherence, and fidelity

We will carefully assess acceptability, safety, adherence, and fidelity of the SELFIE intervention. Participants in the experimental condition will be asked to complete a debriefing questionnaire, which assesses acceptability, satisfaction, and whether or not there were beneficial effects of the EMI tasks and sessions. Also the Working Alliance Inventory (WAI) [96] will be completed by the participant and the SELFIE therapist providing the SELFIE intervention. Adherence to the intervention will be assessed using the implicit EMI adherence data recorded by the app (e.g., number and duration of completed EMI interactive, enhancing and consolidating tasks). Further, the attended face-to-face sessions will be audio recorded and adherence will be rated on a visual analog scale (ranging from 0 = “not at all” to 11 = “very much”) by a clinical psychologist or researchers (supervised by a clinical psychologist).

#### Other measures

A socio-demographic schedule will be used to assess basic socio-demographic and clinical characteristics including age, gender, employment status, and level of education. Resilience will be assessed with the Temperament and Characteristics Inventory (TCI) [97]. Last, other confounders, such as alcohol and substance use (Composite International Diagnostic Interview (CIDI), sections B, J, and L) [98], medication use, treatment classification, and social support (Childhood Experience of Care and Abuse (CECA), section social support) [67], will also be assessed.

### Sample size

Previous studies demonstrated that third-wave cognitive behavioral therapy (CBT) [22, 99, 100], including CBT focusing on self-esteem [51, 101], may lead to reductions in symptoms of psychopathology of moderate to large effect size. In line with previous research, the power calculation is based on the primary outcome of level of self-esteem as measured with the RSES [51]. Power simulation in the R environment indicated that a sample size of 130 participants (65 per condition) is sufficient to test our primary hypothesis of the effect of condition (SELFIE + TAU vs. TAU) on self-esteem, while controlling for self-esteem at baseline. Specifically, this will allow us to detect an effect size (standardized mean difference (SMD)) of 0.3 (experimental vs. control condition), i.e., a difference that is considered clinically relevant, at (at least) post-intervention or 6-month follow-up with a power of 0.87 (primary hypothesis), and, at long term, (at least) at one of the post-intervention and follow-up time points (6-month, 18-month, and 24-month follow-up), with a power of 0.82 when testing at alpha = 0.05 using linear mixed modeling. Based on our previous and ongoing work, we will allow for a 25% attrition rate at 2-year follow-up, which will result in a loss to follow-up of around 22 individuals per condition on average (see Fig. 1). Hence, we will recruit a total sample of 174 participants (87 experimental, 87 control condition) at baseline.

### Randomization and blinding

Each participant will be randomized at a 50:50 ratio to the experimental or control condition after completing the baseline assessment. Randomization will be conducted through a computer-generated sequence, stratified by region of a collaborating center or as external admission. The assessors will be blind to the allocation of subjects when assessing participants at post-intervention, 6-month, 18-month, and 24-month follow-up. After random allocation to the experimental condition, the names and contact details of the participants will be passed on to the SELFIE-therapist providing the SELFIE intervention. This will be done through an independent researcher. This researcher will inform the assessors when assessments at post-intervention and follow-up need to take place for each individual participant. The design of this study is single blinded, because SELFIE therapists and patients cannot be masked towards the allocation of patients to the experimental or control condition. Any data specific to the intervention condition (e.g., on treatment fidelity) will be stored in a separate database. Any breaks in masking will be documented in the trial master file and another assessor will be allocated to complete the next set of assessments where possible.

### Assessment of safety

Serious adverse events (SAE), which include any serious incidents that result in death, persistent or significant disability or incapacity, require (extension of) hospitalization or are life threatening, will be monitored and collected throughout the study period. In case of occurrence, SAEs will be reported to the accredited Medical Ethics Review Committee (MERC), the Data Monitoring and Ethics Committee (DMEC), and the Trial Steering Committee (TSC). While carefully documented, it is not expected that any SAE will occur as a result of the intervention. The DMEC will advise on any ethical or safety concerns, monitor evidence for intervention harm (e.g., SAEs) for the experimental condition, and review whether these events are in line with expectations. If deemed necessary, the DMEC can recommend to the Coordinator and TSC for interim analyses to be conducted and the trial to be terminated prematurely. All reported (serious) adverse events will be reported in publications of findings from this study.

### Statistical analysis

A full statistical analysis plan will be written and published prior to unblinding of the study and before any analysis is being undertaken. The trial data set will be accessed by the investigators to test the primary hypothesis of an improvement in self-esteem at post-intervention and 6-month follow-up in a priori planned statistical analysis when data collection for assessments at 6-month follow-up has been completed while retaining masking of assessors until the last assessment of the last participant at 24-month follow-up. We will use a linear regression model with the primary outcome of self-esteem at post-intervention and 6-month follow-up entered as the dependent variable and self-esteem measured at baseline, condition (SELFIE + TAU vs. TAU), time (as a two-level factor), center (as a four-level factor), the baseline × time interaction, and a time × condition interaction term as independent variables, in line with the intention-to-treat principle. All randomized participants will be included in the analysis and will be analyzed according to the intention to treat principle. Residuals within subjects will be allowed to be correlated with a completely unstructured variance-covariance matrix to take within-subject clustering of repeated measures into account. We will fit the model using Restricted Maximum Likelihood (REML [102]) in Stata 15 [103], which allows for all available data to be used assuming that data is missing at random if all variables associated with missing values are included in the model [104, 105]. Therefore, potential bias due to attrition over the study period, differences between centers, or as a function of baseline self-esteem will be minimized by the model. We will make every effort to assess all participants at post-intervention and follow-up. To test the main effect of condition, an omnibus test of no difference between the two conditions at all two time points (Wald-type test with df = 2 and alpha = .05) will be used. The two time-specific contrasts will be examined if the omnibus test is statistically significant to determine at which time points significant differences are present (each tested at alpha = .05). The two time-specific contrasts (to determine at which time points significant differences are present) will only be examined if the omnibus test is significant and, hence, the family-wise type I error rate of finding at least one significant difference at the three time points is controlled at alpha = .05. Hypotheses in relation to secondary outcomes of momentary self-esteem, positive and negative schematic beliefs of self, resilience, emotional well-being, general psychopathology, functioning, and quality of life at post-intervention and 6-month follow-up will be tested following the same steps. The investigators will access the trial data set to test hypotheses in relation to all four time points (i.e., post-intervention, 6-month, 18-month, and 24-month follow-up) in a priori planned statistical analysis when data collection for assessments at 24-month follow-up has been completed. For hypotheses in relation to primary and secondary outcomes at all four time points (i.e., post-intervention, 6-month, 18-month, and 24-month follow-up), the main effect of condition will be tested using, again, an omnibus test of no difference between the two groups at all four time points (Wald-type test with df = 4 and alpha = .05). The four time-specific contrasts will be examined to determine at which time points significant differences are present (each tested at alpha = .05), if the omnibus test shows to be statistically significant. Since randomization will be performed in blocks, stratified by region of collaborating center or as external admission, all analyses will include this as a covariate, even though it is not expected this variable will lead to bias. As participants will be randomly assigned to experimental and control condition, no differences across conditions are expected in other study parameters (socio-demographics, alcohol and substance use, medication use, treatment classification, social support and self-compassion). If, however, in contrast to what would be expected, there are significant differences at baseline in any of these parameters across conditions, these will be included as covariate(s) in analyses with primary and secondary outcomes as dependent variable. As ESM data have a multilevel structure, multiple ESM observations (level 1) will be treated as nested within time points (i.e., baseline, post-intervention and 6-month, 18-month, and 24-month follow-up) (level 2) and time points will be treated as nested within subjects (level 3).

Cost-effectiveness analysis (CEA) will be conducted based on service use and cost data collected using the TiC-P. Cost-utility analysis (CUA) will be conducted using quality-adjusted life years (QALYs), which will be calculated based on the EQ-5D-5L. For both CEA and CUA, the incremental cost-effectiveness ratio (ICER) will be calculated, which reflects the extra cost needed (or saved) per one unit increase in self-esteem or QALY gained, respectively.

Descriptive statistics will be used and confidence intervals constructed as appropriate to compute basic sample characteristics and summarize findings on acceptability, safety, and intervention fidelity of, as well as adherence to the intervention.

#### Interim analyses and stopping guidelines

Since it is not expected that any harm will occur related to participation in this study, there are no predefined stopping guidelines and no a priori planned interim analyses. The DMEC can recommend to the Coordinator and TSC for interim analyses to be conducted if deemed necessary because of any ethical or safety concerns.

### Research governance

Maastricht University is the sponsor of this study. The trial has received ethical approval from the Medical Ethics Review Committee (MERC) at Maastricht University Medical Centre (MUMC), the Netherlands (reference: NL64393.068.17). Amendments to the study protocol will be submitted to the MERC for approval, then communicated to all relevant parties (DMEC, TSC, the sponsor, funder, and collaborating centers) and will be updated in the clinical trial registry. In case of deviations from the study protocol, a breach report form will be used for documentation. The handling of the data will be in compliance with the Dutch and European General Data Protection Regulation (GDPR). If a participant withdraws their consent, all data from that participant will be destroyed. No biological specimens will be collected in this trial. All data will be handled confidentially and will be coded using a number according the order of entry. In line with the GDPR, all data will be securely stored and personal data will be stored separately from the number-coded data. Consistent with the consortium agreement of this study, the coordinator will have overall responsibility for the trial and will be responsible for the day-to-day management of the project. The project leader advices on, and supports, the coordinator in the day-to-day management of the project. Each party (i.e., School for Mental Health and Neuroscience, Mondriaan, Levvel, Academic Medical Centre Amsterdam, Parnassia) appoints its lead scientist on the project as principal investigator (PI). The coordinator and project leader will liaise closely with all PIs on recruitment and consent procedures. The Trial Management Committee will meet monthly and includes the coordinator, the project leader and all PIs. It will be chaired by the coordinator and will manage the day-to-day running of the study, audit the trial conduct, and oversee preparation of reports to the MERC, the TSC, and the DMEC. The coordinator will permit trial-related monitoring, audits, and MERC review (conducted by the Clinical Trial Center Maastricht, which is independent from the study sponsor (i.e., Maastricht University)). The TSC will meet at least annually to provide independent overall supervision of the trial, to approve the protocol and any amendments and to monitor progress (e.g., data completion rates and adherence to the protocol). Also, the DMEC will meet at least annually. The DMEC will advise on ethical or safety concerns and, for the experimental condition, monitor evidence for intervention harm (e.g., SAEs) and review whether these events are in line with expectations. The DMEC can recommend to the coordinator and TSC to be given access to all trial data as well for interim analyses to be conducted and the trial to be terminated prematurely if deemed necessary.

## Discussion

Exposure to childhood adversity may have deleterious effects on self-esteem, which, in turn, has been shown to be an important putative transdiagnostic mechanism in pathways from childhood adversity to adult psychopathology [37, 38] and, thus, is a promising target for early intervention. Even though self-esteem is a common target of conventional psychological interventions [51, 71, 72, 101], current psychological help remains difficult to access for youth in real-world service delivery settings [49, 50], and therefore, new approaches are required. The current paper presents the study protocol of a multi-center RCT to evaluate the efficacy of an EMI (SELFIE) to improve self-esteem in youth exposed to childhood adversity. SELFIE, an intervention that extends beyond or even outside the clinical setting, has been designed to improve the accessibility and efficacy of psychological interventions for youth exposed to childhood adversity [49, 50]. The potential effects of the SELFIE intervention may help to minimize the deleterious impact of, and hence, resilience to, childhood adversity by improving self-esteem and, thereby, prevent the development of severe and enduring mental disorder later in life and reduce disease burden. This study contains several unique and novel aspects. To our knowledge, SELFIE is the first transdiagnostic EMI that focuses on improving self-esteem in youth exposed to childhood adversity, which will inform our understanding of self-esteem as a psychological mechanism as well as the growing knowledge of mHealth intervention development and implementation, in particular for EMIs. An advantage of EMIs is that the intervention components are delivered in, and therefore more easily translated to, diverse contexts of daily life [54]. In doing so, the SELFIE intervention focuses on positive rather than negative self-esteem, that is, the goal of SELFIE is to build a competing positive self-esteem, without directly targeting more deeply rooted negative self-esteem [72]. This makes this low-level intervention suitable as a guided self-help EMI that is easily accessible, individually tailored and offered in daily life. Also, the multi-center RCT design implemented in different regions of the Netherlands will provide high external validity of findings. Cost effectiveness and cost utility will inform implementation, and the process evaluation on acceptability, treatment adherence, and treatment fidelity will provide important data on potential barriers, but also on potential facilitators for implementation.

## Trial status

The trial has been registered at trialregister.nl (no. NTR 7475) in November 2018, and all study procedures were approved by the MERC at MUMC in August 2018. We are currently working with protocol version 10, originating from February 2021. Recruitment started in December 2018, the first enrollment was in January 2019, recruitment was completed in June 2021, and outcome assessment will continue until December 2022.

## Data Availability

The data resulting from the current study will not be publicly available. The data, protocol, statistical code, and a list of study sites are available from the corresponding author on reasonable request.

## Abbreviations

AE: Adverse event
BCSS: Brief Core Schema Scales
BPRS: Brief Psychiatric Rating Scale
CECA: Childhood Experience of Care and Abuse
CIDI: Composite International Diagnostic Interview
CTQ: Childhood Trauma Questionnaire
DMEC: Data Monitoring and Ethics Committee
EMI: Ecological Momentary Intervention
ESM: Experience Sampling Method
EQ-5D-5L: EuroQol – 5 Dimensions – 5 Level version
K10: Kessler Psychological Distress Scale
MERC: Medical Ethics Review Committee
MUMC: Maastricht University Medical Centre
mHealth: Mobile Health
PANAS: Positive and Negative Affect Scale
RCT: Randomized controlled trial
RBQ: Retrospective Bullying Questionnaire
RSES: Rosenberg Self-Esteem Scale
SELFIE: Ecological momentary intervention for improving self-esteem
SERS: Self-Esteem Rating Scale
SOFAS: Social and Occupational Functioning Assessment Scale
TAU: Treatment as usual
TCI: Temperament and Characteristic Inventory
TiC-P: Trimbos Institute and Institute of Medical Technology Assessment Questionnaire for Costs associated with Psychiatric Illness
TSC: Trial Steering Committee
WHOQOL-BREF: World Health Organization Quality of Life Instrument-Brief
